# Methylphenidate as a treatment option for substance use disorder: a transdiagnostic perspective

**DOI:** 10.3389/fpsyt.2023.1208120

**Published:** 2023-08-03

**Authors:** Peter van Ruitenbeek, Luisa Franzen, Natasha Leigh Mason, Peter Stiers, Johannes G. Ramaekers

**Affiliations:** Department of Neuropsychology and Psychopharmacology, Faculty of Psychology and Neuroscience, Maastricht University, Maastricht, Netherlands

**Keywords:** addiction, attention deficit hyperactivity disorder, psychopharmacology, methylphenidate (MPH), cognitive control

## Abstract

A transition in viewing mental disorders from conditions defined as a set of unique characteristics to one of the quantitative variations on a collection of dimensions allows overlap between disorders. The overlap can be utilized to extend to treatment approaches. Here, we consider the overlap between attention-deficit/hyperactivity disorder and substance use disorder to probe the suitability to use methylphenidate as a treatment for substance use disorder. Both disorders are characterized by maladaptive goal-directed behavior, impaired cognitive control, hyperactive phasic dopaminergic neurotransmission in the striatum, prefrontal hypoactivation, and reduced frontal cortex gray matter volume/density. In addition, methylphenidate has been shown to improve cognitive control and normalize associated brain activation in substance use disorder patients and clinical trials have found methylphenidate to improve clinical outcomes. Despite the theoretical basis and promising, but preliminary, outcomes, many questions remain unanswered. Most prominent is whether all patients who are addicted to different substances may equally profit from methylphenidate treatment.

## 1. Introduction

Substance use disorder (SUD) is currently one of the most prominent mental disorders worldwide (1). According to the “World Drug Report” from 2019 by the United Nations Office on Drugs and Crime, around 36 million people suffer from SUD, which may be an underestimation, given that estimates for Europe reached 15 million cases of alcohol dependence alone in 2011 (2). The large scale of SUD occurrence bears an enormous burden to many individuals and society as a whole (3). For example, the current opioid epidemic leads to a rapidly increasing number of overdose deaths due to opioid misuse (4), and out of all brain disorders, alcohol use disorder is estimated to induce the third highest number of years of life lost (2). These numbers highlight the need for successful treatment of patients with SUD.

Existing treatments of SUD often fail to prevent relapse, and progress has been modest over the last 20 years. Only 17–35% of the individuals treated for alcohol use disorder (including pharmacotherapy, cognitive behavioral therapy, and group sessions) stayed abstinent for at least 1 year as reported in 2001 (5), while in 2016 a conservative estimate of 35% was obtained of SUD patients who can be considered in long-term remission (6). In addition, only a minority of SUD patients receive treatment. Development of effective prevention and cost-efficient novel treatment programs for SUD are, therefore, of significant importance, and alternative approaches should be explored.

New treatment approaches may stem from novel approaches to diagnose psychopathology. Specifically, the traditional categorical approach to psychopathology attempts to identify treatments based on maladaptive behavior and symptoms that characterize a disorder in a narrow sense by intentionally excluding overlap with other disorders. More concretely, a diagnosis for a given disorder is less likely when symptoms can be explained by another disorder. Therefore, the approach largely ignores behavioral and neural deficits shared by different disorders (7), thus potentially overlooking effective treatments. In contrast, contemporary approaches to diagnoses of mental disorders utilize various dimensions of symptoms within and across disorders. For example, deficits in executive functions may be characteristic of multiple mental disorders such as attention-deficit hyperactivity disorder (ADHD) and SUD [but also, among others, schizophrenia, autism, and Alzheimer's disease (1)]. This view is becoming more prevalent as it is acknowledged in the current version of the Diagnostic and Statistical Manual of Mental Disorders [DSM-5; (1)]. Nonetheless, the DSM-5 can still be considered to be in a transitional period, as it does not fully endorse the view. Others take the dimensional approach further. For instance, the Research Domain Criteria project [RDoC; (8)] classifies disorders as quantitative variations on dimensions of a set of constructs (e.g., cognitive control) within domains (e.g., cognitive systems). These constructs are defined by elements that include behavioral performance, self-report, and biological mechanisms (c.f. https://www.nimh.nih.gov/research/research-funded-by-nimh/rdoc/constructs/rdoc-matrix). As these constructs can cut across mental disorders, we consider it reasonable that treatments follow suit by exploring treatments for seemingly different disorders that are similar in, at least some, underlying cognitive constructs and associated biological mechanisms. These shared deficits within the constructs can provide important opportunities for treatment (9, 10). Therefore, the potential of a pharmacological treatment to be used in SUD, but which is currently used for a disorder that shows similarities in the construct of cognitive control, should be explored.

Treatments for ADHD may be useful in SUD as both disorders are characterized by poor performance within the construct of cognitive control. The more specific aim of this study was to evaluate the potential of pharmacological ADHD treatment for successful use in SUD. At the core of the idea to utilize pharmacological treatment for ADHD in SUD lies the cognitive control deficit represented by inhibitory control failures as the most prominent (11, 12), which is shared between the two conditions (13) and the underlying dopaminergic dysfunction. In general, inhibitory control is the ability to suppress a prepotent or habitual response when necessary (14). When inhibitory control is lacking, actions are immediately and impulsively executed, often at the expense of long-term consequences, and with the disregard for long-term goals. These actions are inappropriate to the situation and are difficult to terminate once started. On a surface level, poor inhibitory control in SUD is demonstrated by displaying addiction behavior (e.g., consuming drugs), while rationally being aware that it is better not to do so given the long-term consequences (1). Similarly, in ADHD, poor impulse control is expressed by the inability to remain physically immobile and by frequent engagement in distractions (1). On the task-performance level, individuals with ADHD or SUD both perform poorly on laboratory measures of inhibitory control, such as the stop-signal task (SST) (11, 15). Lower response regulatory abilities have also been coined a risk factor for substance abuse (16). On a neurotransmitter level, dopamine (DA) receptor families play a prominent role in cognitive and inhibitory control (17), particularly deficient D2-like receptors are observed in individuals with high impulsivity (18). In addition, both ADHD and SUD patients show abnormal dopaminergic neurotransmission (18–20). Therefore, this transdiagnostic impairment in both ADHD and SUD suggests inhibitory control to be a suitable construct to investigate further as a target for pharmacological treatment (13). The potentially shared underlying neurobiological correlates should be explored to validate the pharmacological treatment. However, it should be noted that other disorders may share the neurobiological atypicalities as well as ADHD and SUD. Those disorders may not be suitable (e.g., schizophrenia) to be treated with typical ADHD medication. Therefore, the rationale presented below should be considered within the boundaries defined by the suitability of disorders to be treated with stimulant medication.

Existing pharmacotherapy for SUD does not appear to effectively target cognitive control (21). Nonetheless, neuropharmacological therapy is the most prominent treatment to target cognitive control deficits and deficient DA transmission in ADHD and is considered effective. DA-based ADHD interventions have a success rate of around 70% (1). While various stimulant drugs exist to treat ADHD, methylphenidate (MPH) is the most widely used and therefore, given the suggested overlap, can be considered a prime candidate for treating SUD. MPH blocks dopamine (DAT) and noradrenaline (NAT) transporters (22) and thereby increases extracellular DA and noradrenaline (NA) levels. MPH improves response inhibition task performance and reduces impulsive behavior in children with ADHD (23). MPH also has been shown to improve inhibitory control abilities in cocaine-dependent patients (24, 25), although this may be due to normalizing catecholamine levels by MPH in these particular patients. In addition, MPH has been considered safe with low addiction liability due to the slower onset and elimination of its effects (26). These findings suggest that MPH may successfully increase inhibitory control in SUD as it does in ADHD.

The purpose of this perspectives paper was to evaluate the potential for methylphenidate to be a suitable treatment option in SUD. By comparing ADHD and SUD on a (a) theoretical level, (b) behavioral/clinical level, and (c) neurobiological level, seemingly similar deficits are explored to assess the stimulant treatment's potential. Second, some direct evidence for the efficacy and effects of MPH in SUD treatment and criticism are reviewed, and finally, a guide is proposed for future research into knowledge gaps, ultimately to establish new treatments for SUD.

## 2. Evaluation of the hypothesis

### 2.1. ADHD and SUD overlap in theoretical models

Both theoretical models of ADHD and SUD attribute a central role to reduced or biased cognitive control of behavior. Currently, one hypothesis for the mechanism underlying ADHD characteristic dysfunction is that low tonic catecholamine (DA, NE) activation leads to phasic hyper-responses causing distractibility, impulsivity, and disorganized thoughts (19, 27). ADHD is also associated with low prefrontal cortex (PFC) functioning, which causes reduced control over both external and internal stimuli, and reduced control of behavior. The PFC controls stimuli by dynamically inhibiting sensory cortices and subcortical structures. In ADHD, both structure and function of PFC are affected, reducing the ability to regulate information, which results in distractibility and impulsivity (28). This notion of core deficits in ADHD is supported by observations that the PFC, caudate, and cerebellum as a network are most prominently affected in ADHD (29, 30).

SUD is currently best explained by extended hypotheses that emphasize the increased value of potential rewards (31, 32) and increased habitual control of behavior (33, 34). The incentive salience/sensitization hypothesis states that stimuli that predict drug consumption cause an intense “wanting” of the drug, which is associated with a hyper-responsive mesolimbic DA system (31, 32). This response amplifies the motivational drive to obtain the predicted drug at the expense of long-term consequences and can, therefore, be considered biased goal-directed behavior. A second hypothesis that attempts to explain addiction behavior stresses reduced control over behavior, rendering goal-directed behavior habitual (33, 34). This transition is marked by a shift in brain activation from ventral striatum, indicating reward-based goal-directed behavior, to dorsal striatum, indicating fast, non-controlled behavior. While these hypotheses appear to differ in the way goal-directed behavior is affected (biased in incentive salience and reduced in goal-directed to habitual shift), both share a notion of a reduction in the weight of the behavioral choice to refrain from drug taking and thereby a relative lack of cognitive control directed at achieving long-term goals.

Taken together, both conditions are theorized to implicate insufficient or biased behavioral control governed by phasic catecholamine responses.

### 2.2. ADHD and SUD shared behavioral deficits

Impaired cognitive control as a broad term is often operationalized as various measurements of impulsivity. For example, the BIS-11 is an established self-report measurement of impulsivity (35). The scale consists of different subscales capturing aspects of inattention, spontaneous actions, lack of forethought, non-planning, and inhibition (36). More objective measures of impulsivity are provided by perseveration paradigms [e.g., Kertesz et al. (37)], antisaccade paradigms (38), conflicting or contralateral motor response tasks [e.g., Bellato et al. (39), Watson et al. (40)], go/no-go tasks [e.g., Trommer et al. (41)], and the stop-signal paradigm (42). All paradigms aim to assess the ability to inhibit prepotent responses. Antisaccade paradigms, conflicting and contralateral motor response tasks, go/no-go, and perseveration tasks all require the participant to withhold or change a response to a stimulus, whether the response is a saccade, eye-gaze, or a simple motor response like a button press. Perseveration paradigms are characterized by the need to change a response from a previously given response. Antisaccade paradigms require participants to make goal-directed saccades in the opposite direction to a presented stimulus, which elicits a reflexive saccade that needs to be suppressed. Similarly, a conflicting or contralateral motor response task requires the participant to make a spatial non-congruent response to a visual or tactile stimulus, e.g., in opposite direction to an indicated location. The go/no-go paradigm entails responding or withholding a cued response depending on the identity of the presented stimulus. Similarly, in the stop-signal task participants need to respond as fast as possible every time they see a target stimulus within a sequence of other insignificant stimuli (Go trials). However, when the target stimulus is presented together with a secondary cue, participants must refrain from responding (15). The difference between this paradigm and the former is that the secondary cue is presented after the imperative cue and therefore the stop-signal response time (SSRT) can be calculated, which enables quantification of the time needed to inhibit a response (43).

ADHD-diagnosed individuals show higher BIS-11 scores compared with matched controls (44), suggesting increased subjective impulsivity. Objectively assessed impulsivity is also increased in ADHD adults (45) as well as children (46, 47), particularly as assessed with the stop-signal task (46, 48). Up to 1,000 ms, slower inhibition of their responses compared with healthy controls has been observed (49, 50), while recent studies find smaller but significant differences between these groups (51). However, some studies report no group differences (52), which may be explained by the suggested ability of adults with ADHD to compensate for their deficits for a short amount of time (53). ADHD patients have shown impaired performance on antisaccade tasks compared with healthy controls, although the results are not unequivocal (54, 55). They also show lower scores on tests of perseveration [e.g., Houghton et al. (56), Fischer et al. (57)], conflicting motor response [e.g., Mahone et al. (58)], and on go/no-go paradigms (59).

Similar to ADHD individuals, SUD patients have shown high scores on the BIS-11 questionnaire indicating enhanced subjective impulsivity levels (60, 61). Various measures of brain structure and functions associated with inhibition failure have been shown to predict binge drinking during adolescence (62). Poor response inhibition abilities have also been shown to predict adolescent drug and alcohol use (63). More specifically concerning the SST, drug-dependent individuals show impaired performance on the SST. For example, individuals with cocaine use disorder have a lower probability of successfully inhibiting a response and do this more slowly compared with healthy controls (11). Furthermore, longer SSRTs can predict the degree of future alcohol consumption and progression toward dependence in heavy drinkers (60). Similarly, increased electrophysiological activation during SST performance predicts smoking cessation duration (64) and smoking behavior (65).

SUD patients have been found to display rigid response behavior by showing increased perseveration in a probabilistic reversal learning paradigm compared with healthy controls (66, 67). In addition, these differences could be reversed using the dopaminergic drugs pramipexole (66) and amisulpride (67). A correlation between perseveration and duration of cocaine use has also been observed using an instrumental learning paradigm (68). However, perseveration did not differ from healthy controls in this particular study (68). In addition, increased perseveration in SUD patients has not been observed consistently across addictions (69). While cocaine users did show perseveration, chronic amphetamine and opioid users did not (70).

Nicotine-dependent individuals have been shown to perform worse compared with healthy controls on go/no-go paradigms (61), which is correlated with how much a person smokes per day (71). The poor performance on go/no-go paradigms has also been associated with relapse vulnerability (72). In addition, satiated smokers show impaired performance monitoring potentially contributing to the continuation of their smoking habits (73).

Adolescents at risk for developing an addiction show reduced antisaccadic performance (74) or a lack of performance improvement during adolescence (75), but which could be enhanced using incentives (76, 77). Performance on antisaccadic eye movements has been found to correlate with smoking status (71). Please see Table 1 for a qualitative summary of the behavioral observations.

**Table 1:** Qualitative summary of differences between patients with attention-deficit/hyperactivity disorder (ADHD) and healthy controls, and between patients with substance use disorder (SUD) and healthy controls on subjective and objective measures of impulsivity.

**Tasks of impulsivity**	**Condition**	
	**ADHD**	**SUD**
BIS-11	+	+
Perseveration	+	+/-
Antisaccade	+	+
Conflicting motor response	+	-
Go/no-go	+	+
Stop signal	+	+

+, presence of impairment; +/-, inconsistent findings; -, no observation of impairment.

To the best of our knowledge, there is one study comparing ADHD and SUD directly on objective measures of inhibitory control. Gerhardt et al. (78) observed more commission errors in ADHD subjects compared with alcohol use disorder subjects in a comprehensive paradigm assessing various cognitive aspects of impulsivity. However, they did not observe any behavioral differences on six other measures. Further direct comparisons that result in differences between the groups may argue against a common deficit. However, as presented above, both populations can be successfully discriminated from healthy controls using the BIS-11 (44, 60). In addition, similar cognitive control performance patterns already provide some objective evidence for similar inhibitory control deficits. Similar neural abnormalities might be responsible for the observed inhibition impairments and may strengthen this position.

### 2.3. ADHD and SUD shared neurobiological characteristics

Altered neurobiological metrics in both ADHD and SUD that form an overlap in brain areas that govern inhibitory control of behavior can be considered evidence for the shared deficits. Shared differences compared with neurotypical and healthy controls in neurotransmission, brain activation, and brain matter volume/density may exist. The aim of the following section is not to provide an exhaustive review of the neurobiological alterations in ADHD and SUD, but identification of overlap relevant to inhibitory control. Given that MPH targets catecholamine neurotransmission, shared catecholamine deficits should have the most weight in the evaluation.

#### 2.3.1. Dopamine

DA plays a major role in the regulation of behavior, and small changes in DA levels impair cognitive control (29). Abnormal DA neurotransmission is one of the most important factors leading to behavioral dysfunction in ADHD (79, 80). Within the PFC–striatal–PFC loop, the “tonic–phasic DA hypothesis” of ADHD offers an explanation for characteristic ADHD symptoms (27, 81, 82). The model describes how a striatal imbalance between D1-like and D2-like receptor activation in patients with ADHD (79) affects the gating of PFC-striatal signals. The reduced gating ability leads to hyperresponsiveness of the individual. According to the model, normal striatal gating function is established by tonic extracellular DA concentrations that activate D2-like autoreceptors. This tonic DA-induced D2 receptor activation subsequently reduces phasic DA responses by a reduction in DA synthesis and release (81). In ADHD-affected individuals, low tonic striatal DA activity leads to decreased inhibition and subsequent increased phasic (burst of high level) DA signaling via striatal postsynaptic D1-like receptor activity (83, 84). Corresponding non-optimal receptor activation balance (19, 84) and low tonic/high phasic activation patterns (19) observed in individuals with ADHD support this view.

Increased DAT activity might underlie the observed D1-like/D2-like receptor activation imbalance (19). Elevated DAT levels accelerate DA reuptake and therefore reduce tonic D2-like receptor activation disinhibiting phasic activity. Multiple studies report heightened DAT levels in individuals with ADHD, which might be the result of inadequate neurodevelopment (23, 85, 86). These findings must be interpreted with caution because other studies do not confirm elevated DAT levels in ADHD patients (87). The inconsistent findings might originate from different inclusion criteria, methods, or screening techniques. Nonetheless, the most successful treatment for ADHD, MPH, blocks the DAT (88). DAT occupancy by MPH is positively correlated with reduced self-reported impulsivity (89). Therefore, whatever the neural deficit in ADHD, targeting DAT appears successful in ADHD treatment.

Taken together, while it is still unclear whether poor PFC functioning is the cause of low tonic DA activation in the striatum, and what role DAT activity plays in this, or whether increased phasic striatal output causes poor PFC functioning, it is clear that this PFC DA circuitry plays a prominent role in ADHD-related behavioral deficits (80). In favor of this view, stimulant-induced enhancement of catecholamine function in the PFC of ADHD individuals is associated with behavioral improvements and can be reversed by noradrenergic α2 and DA D1 receptor antagonists (80).

Alterations in dopaminergic functioning have been observed in alcohol, cocaine, opioid, cannabis, and tobacco-addicted individuals (24, 90, 91) and have been well-studied using PET and SPECT (92). Drugs elicit high DA surges in the mesolimbic reward system (93). Tonic DA activity is suggested to be reduced to compensate for these excessive DA responses [(94), p. 104]. Cannabis users have shown reduced DA synthesis (95) and reduced striatal DA response following a stimulant challenge (96, 97), which might explain the lost interest for natural rewards and compulsive drug-seeking in SUD patients. Conversely, increased DA signaling has been observed subsequent to the presentation of addiction-related stimuli (98, 99) and D2/D3 antagonism reduced cue-induced responding and reward-obtaining impulsivity (100). In addition, reduced D2-like receptor availability has been observed (18, 91, 101), which correlates with age of cannabis use onset (97) and current use history (102). In addition, lower D2 receptor availability in the dorsal striatum in methamphetamine users predicted relapse (103). Lower D2-like receptor levels might underlie the observed hypofrontality and control impairments in SUD patients (104). A study showed that blocking D2-like receptors decreases prefrontal activity, especially in the IFG and anterior cingulate cortex (ACC), compared with participants receiving a placebo (104). The attenuated brain activation correlated with performance impairments in an SST. These findings confirm the regulatory function of DA in prefrontal inhibitory control mechanisms.

Concerning DAT availability, the many studies performed report inconsistent findings in SUD patients. Researchers argue for unchanged (105), increased (106), or decreased DAT densities (107, 108) in this population. Reduced DAT availability might be a reversible neuroadaptive response to the attenuated tonic DA activity observed in drug-dependent individuals (107, 109). When tonic DA levels are low, DAT may be downregulated to accommodate sufficient DA activity despite the lower levels. Contrariwise, increased DAT levels might be a failed attempt to compensate for extremely high DA levels following drug binges (23). It is of great clinical relevance to clarify DAT's role in addiction as many drugs, such as MPH, target these molecular complexes (93).

In conclusion, both ADHD and SUD are characterized by a hyperresponsive mesocortical DA system exerting increased phasic responding upon relevant (e.g., addiction associated) stimulation, which may be associated with reduced D2-like receptor function, low tonic DA, and altered DAT activity. However, findings concerning DAT levels are inconsistent for both conditions. For ADHD, the evidence appears to lean toward increased DAT levels whereas for SUD evidence points equally in both directions. Importantly, the mechanisms underlying the disorders differ, such that in SUD there may be an overall reduction in DA functioning leading to low tonic DA activation and low phasic activation in response to natural rewards, while only displaying high phasic activation to addiction-related stimuli. Conversely, in ADHD the low tonic activation is hypothesized to lead to high phasic activation toward a large number of different stimuli.

#### 2.3.2. Brain activation

##### 2.3.2.1. Mesolimbic system

The mesolimbic system plays a pivotal role in reward-based learning and incentive salience (110) and includes, most importantly, DA neurons in the ventral tegmental area and projections toward the nucleus accumbens. Exaggerated mesolimbic activity has been observed in animal models in which rats exhibit ADHD symptoms (111). However, only a few studies propose a hyperactive mesolimbic system in human individuals with ADHD, for example, when monetary rewards are presented to participants (112). Therefore, further research needs to clarify whether these functional deviations exist in human ADHD populations.

Mesolimbic neuroadaptations in SUD patients have been observed more frequently. Particularly relevant is the hyperactive mesolimbic reward system when addicted individuals are presented with drug-related cues [(113–115), see Leyton and Vezina (116); Berridge and Robinson (31) for nuanced views]. For example, alcohol-dependent drinkers show greater striatal activation in response to alcohol-related cues compared with social drinkers [(117), but see Vollstadt-Klein et al. (118) for conflicting results]. Following addiction-relevant cues, cocaine users (119), cannabis users (120, 121), alcohol-dependent patients (122–124), smokers [(125–127), but see Vollstadt-Klein et al. (128)], and cannabis users and heavy alcohol drinkers (129) all show increased frontostriatal activation (most often including nucleus accumbens) compared with neutral cues. The striatal response has also been associated with alcohol use problems (130) and, among other factors, the amount of alcohol used (131). This may reflect increased signaling of potentially high reward value and subsequent high motivation to obtain the substance of abuse (31). The high motivational drive may not be appropriately governed by frontal cortex circuits and ultimately leads to behavior strongly biased toward obtaining the substance.

Altogether, both ADHD and SUD individuals may show maladaptive mesolimbic processes. The high phasic DA-dependent striatal responses to environmental cues signaling potential rewards may lead to impulsive behavior. Ultimately, these maladaptive neuronal characteristics impair ADHD and SUD patients in their inhibitory control abilities and goal-directed behavior.

##### 2.3.2.2. Cognitive control-related areas

Cognitive control is mainly mediated by an interaction between a frontoparietal network (14) and subcortical structures (132) in which DA plays a prominent role (133). Numerous dopaminergic connections between the subcortical areas and the PFC allow the inhibition of prepotent impulses (134). For example, frontal cortical “top-down” mechanisms inhibit subcortical “bottom-up” impulses via reciprocal connections (135), and the basal ganglia (e.g., striatum, subthalamic nucleus) can facilitate or inhibit frontal processes and therefore modulate behavior (136). Lesions in frontal areas can increase impulsive and disinhibited behavior (137), which is shown by several studies suggesting an association between frontal lobe functioning and performance on a response inhibition task (136, 138). Therefore, impulsive–compulsive disorders may be associated with frontal lobe functioning, which may occur in both ADHD and SUD (109). As a number of studies report hypofrontality in both populations (136, 139), the extent of hypofrontality in both disorders may suggest a common brain-function deficit.

Functional imaging robustly supports prefrontal hypoactivity in individuals with ADHD. ADHD patients show attenuated activity in the ACC and dorsolateral prefrontal cortex (DLPFC), during cognitive task performance (140–142) and significant hypoactivity in inferior prefrontal and orbitofrontal cortices (OFC), striatum, thalamus, and parietal cortices (141). Decreased PFC activity is particularly evident in the performance of tasks that require sustained attention or inhibition of inappropriate movement (143). In addition, in an electrophysiological study, ADHD patients showed reduced activity in the superior frontal gyrus, which modulates self-control during the performance of a cognitive task, compared with controls (51). In support, transcranial direct current stimulation of the DLPFC can improve inhibitory control and reduces impulsivity in ADHD patients (140). That said, not all studies confirm these activation differences. For example, Dillo et al. (53) reported no prefrontal hypoactivity, but increased recruitment of attentional parietal areas. However, these findings may reflect less-efficient processing or compensatory strategies (52, 144) indicating non-optimal functioning.

From a brain network perspective, many of the brain areas that have been found hypoactive are part of the executive control network [ECN, (145)] consisting of the inferior frontal gyrus (IFG), ACC, pre-supplementary motor area (pre-SMA), DLPFC, anterior insula, and posterior parietal cortex (146–148). The ECN is hypoactive in individuals with ADHD during cognitive tasks (149). In addition, the PFC is less extensively connected with subcortical regions (139), which may lead to insufficient 'top-down' regulation of the default mode network [DMN, (150)] and the dorsal attention network (145). This was confirmed by observed disrupted functional connectivity between prefrontal control areas and regions of the DMN (151). In addition, task engagement should decrease DMN activation, but in children with ADHD, the network is activated during an inhibitory control task (52, 152). The disrupted interplay between the ECN and DMN might explain deficient control abilities in individuals with ADHD.

Frontal hypoactivation is also observed in SUD [(136), see Klugah-Brown et al. (153) for a meta-analysis]. For example, cocaine-dependent participants can be discriminated from healthy controls based on attenuated frontal activity during an SST (154). Especially areas of the ECN are affected, including, but not limited to, the IFG, ACC, and DLPFC (155, 156). Methamphetamine users showed reduced activation in brain areas associated with cognitive control (right IFG, supplementary motor area/ACC, and anterior insula) and performed worse than controls on a Stroop task (157). In further support, exciting the hypoactive DLPFC in SUD patients can improve decision-making and decreases craving (155). Furthermore, metabolism in the ACC and OFC is attenuated in individuals with SUD (155, 158), potentially contributing to reduced sensitivity to negative consequences of to-be-performed behavior (155, 159). However, these findings may reflect a neuronal vulnerability as deficient inhibitory control processes have been observed in non-consuming biological siblings as well as drug-dependent individuals (160), and white matter abnormalities are shared by first-degree biological relatives of SUD patients, who have no history of drug use (136).

One study directly compared brain activation of ADHD and alcohol use disorder patients as elicited by a comprehensive response inhibition task (78). Authors report more activation of a frontoparietal network, cortical and subcortical motor areas, and occipital areas in alcohol use disorder patients compared with ADHD patients. Taken together, while some activation differences seem to exist between SUD and ADHD patients, in comparison with healthy controls the hypoactivation of the ECN, and in particular the DLPFC, ACC, OFC, and inferior frontal gyrus, and the increase in cognitive control following DLPFC stimulation are shared between ADHD patients and SUD patients.

#### 2.3.3. Gray matter

In addition to reduced activation, several imaging studies have shown that the DLPFC is smaller in patients with ADHD compared with controls (151, 161–164). Despite that whole brain reduction in thickness, volume, folding, and surface area have been observed (165), suggesting non-specific brain deficits, various measures converge on PFC deformation (28). Variations in dopamine D4 receptors (166, 167) are associated with thinning of PFC in ADHD (168) and reduction in DLPFC neuron density (169). In addition, PFC maturation has been observed to be slower in ADHD (167). Nonetheless, smaller caudate/putamen volume appears most prominent across various meta-analyses (28). These results support the earlier discussed potentially maladaptive prominent PFC–striatal–PFC network characteristics in ADHD patients.

In line with the idea of reduced cognitive control in SUD are the volumetric differences with healthy controls. A recent meta-analysis of 60 voxel-based morphometry studies shows reduced volume of the ACC, thalamus, and insula and increased putamen volume (170). This pattern of results is possibly reflective of decreased cognitive control and increased putamen (i.e., habits) governance of behavior. In addition, a large-scale analysis of gray matter volume in SUD patients has shown brainwide reduction in cortical thickness. While results were mostly driven by alcohol-dependent patients, thinning of OFC, inferior parietal, insula, and middle temporal cortices is shared across addictions to various substances (171). OFC plays an important role in value assignment to future rewards (172), damage to which leads to poor decision-making (173) and may help explain substance-biased behavior.

**Figure 1:**
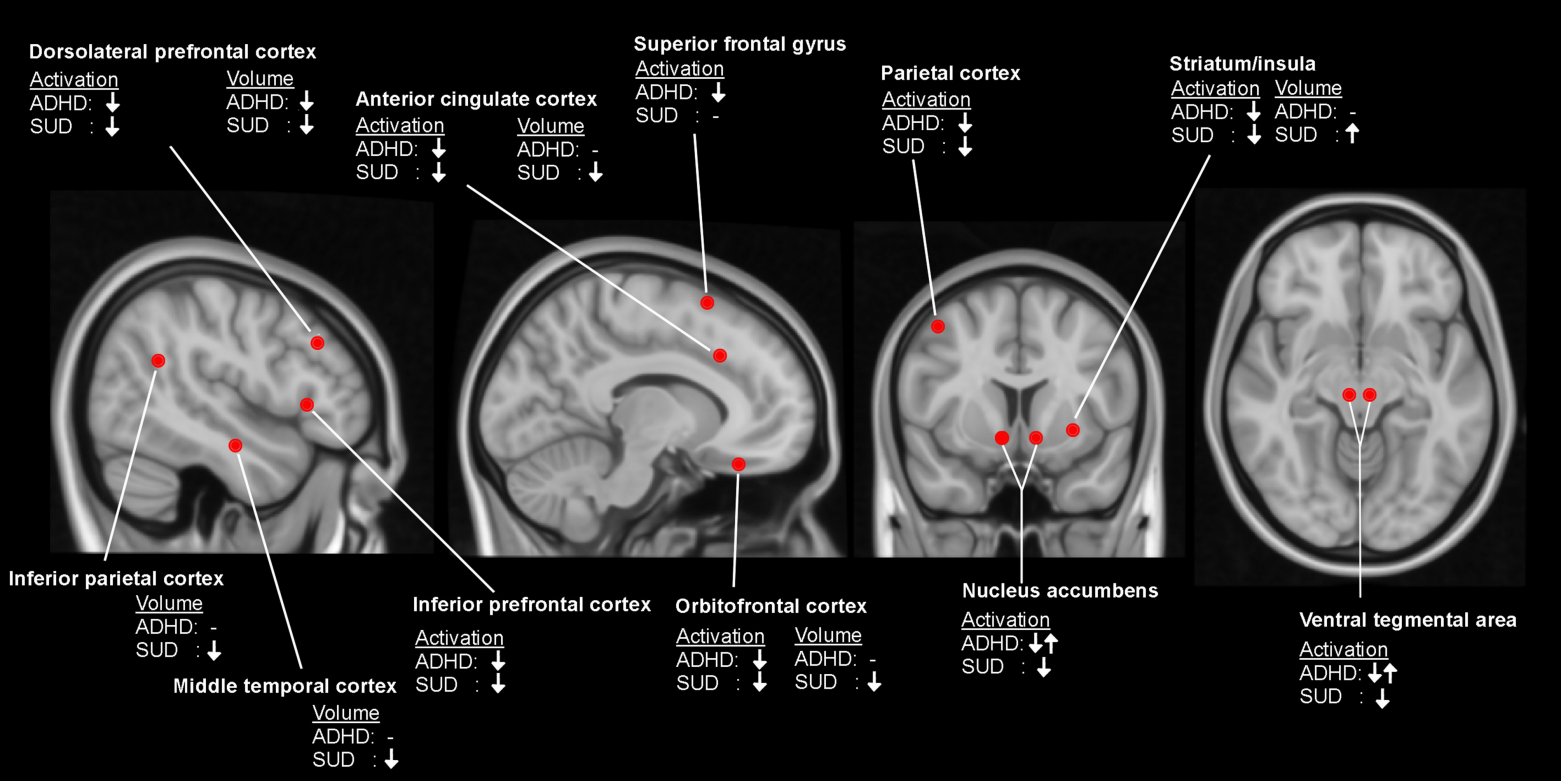
Qualitative overview of differences between patients with attention-deficit/hyperactivity disorder (ADHD) and healthy controls, and between patients with substance use disorder (SUD) and healthy controls in brain activation during performance of cognitive control tasks and measurements of local brain volume. ↓, reduced activation/volume; ↓↑, inconsistent findings; -, no observed difference.

In summary, evidence for gray matter abnormalities in ADHD most strongly points to reduced DLPFC, while SUD appears best characterized by abnormalities of the OFC. Nonetheless, both structures are part of an executive control system governing reward-motivated behavior (174). ADHD and SUD appear to be differentiated concerning putamen volume, where smaller volume is observed in ADHD and larger in SUD. Please see Figure 1 for a qualitative overview of structural and functional differences between ADHD patients and healthy controls and between SUD patients and healthy controls.

### 2.4. Current evidence for MPH efficacy in SUD

Studies aimed at temporarily reversing neurobiological and behavioral deficits in SUD patients largely show that MPH is able to normalize brain function and task performance associated with three key deficits. First, concerning impulsivity, MPH has been shown to decrease stop-signal reaction time (SSRT) in cocaine users which correlated positively with middle FC activation and negatively with ventral medial PFC (25). A complementary analysis of the same data showed that MPH also restored [otherwise impaired (175)] activation in the ventral medial PFC before making commission errors on the stop-signal task (176). Similar findings of normalizing effects of MPH in cocaine users were observed for a cue reactivity task and ACC activation (24, 177, 178). Second, MPH improved cognitive control as shown by increased performance in both cocaine SUD patients and healthy controls on the Stroop task and selectively increasing DLPFC activation in SUD patients (179). Finally, hyperresponsiveness to drug-related cues was reduced by MPH in cocaine users (180).

Direct evidence for the efficacy of stimulants as treatment for SUD has been reviewed a number of times in the recent past. From these reviews emerges a view that MPH is the most, and perhaps the only (181), effective stimulant treatment [however see Dursteler et al. (182) for a nuanced view]. Two studies have shown higher abstinence rates from methamphetamine as a primary outcome after subchronic MPH administration compared with placebo [10–22 weeks; (183–185), but see Miles, Sheridan et al. (184)], as reviewed in Soares and Pereira (186). Ling, Chang et al. (183) observed approximately 15% positive urine drug screens after 14 weeks of MPH treatment vs. ~34% for placebo. Moreso, in another but similar trial, the number of positive urine drug screens was also lower (~20%) compared with placebo (~35%) after 10 weeks of MPH treatment (185) or showed a reduced probability of being positive (187). In addition, MPH showed lower scores on measures of depression and craving (185), withdrawal symptoms, and addiction severity as secondary outcomes (186). Next to these studies, two other studies have shown favorable treatment outcomes of MPH in a sustained release formulation [(188, 189), reviewed in Lee et al. (190)]. After 10 weeks of MPH treatment, 46% of the urine tests were positive for the presence of amphetamine compared with 79% after placebo treatment (188). Finally, adding MPH to an existing behavioral therapy was superior in reducing craving and addiction severity, increasing mental health and number of negative urine tests than either treatments alone (191). Although MPH treatment remains to be refined (e.g., establishing dose–response curves in various SUD patient groups, with comorbidities, and with various addiction severities), these findings strongly suggest the potential value of MPH treatment. Please see Table 2 for an overview.

**Table 2:** Qualitative overview of studies assessing the efficacy of methylphenidate treatment for substance use disorder.

**References**	**MPH treatment**	**Primary outcomes. Executive functions/brain activation**	**Reported additional/side effects**	**Support**
Goldstein et al. (24)	20 mg p.o.	Normalized ACC activation in cocaine users	- commission errors: ↓ - sleepiness: ↓ - performance confidence: ↑ - distrustfulness: ↓ - heart rate: ↑ - systolic and diastolic blood pressure: ↑	Yes
Goldstein and Volkow (178)	20 mg p.o.	Decreased commission errors on Stroop task, increased cdACC, rvACC/mOFC	- craving: ↓↑	Yes
Li et al. (25)	0.5 mg/kg i.v.	Decreased SSRT and restored brain activation in cocaine users.	- heart rate: ↑ - systolic and diastolic blood pressure: ↑ - euphoria: ↑ - anxiety: ↑ - cocaine craving: ↑	Yes
Matuskey et al. (176)	0.5 mg/kg i.v.	Restored brain activation in cocaine users	- heart rate: ↑ - systolic and diastolic blood pressure: ↑	Yes
Moeller et al. (179)	20 mg p.o.	Increased performance on Stroop task and increased DLPFC activation in cocaine users. Reduced ACC activation	- total errors: ↓ - post error slowing ↑	Yes
Volkow et al. (180)	20 mg p.o.	Reduced hyperresponsiveness of the limbic system to drug-related cues in cocaine users	- craving: ↓↑ - heart rate: ↑ - systolic blood pressure: ↑	Yes
		Abstinence		
Aryan et al. (191)	Month 1: 10 mg/day p.o.; Month 2: 7.5 mg/day p.o.; Month 3: 5 mg/day p.o.	Combined MPH and matrix Model treatment increased negative methamphetamine urine tests	- mental health: ↑ - craving: ↓ - addiction severity: ↓	Yes
Dursteler-MacFarland et al. (192)	60 mg/day p.o. for 12 weeks	No difference in negative drug tests	- reported cocaine use: ↓↑ - adverse effects: ↓↑	No
Grabowski et al. (193)	20/25 mg/day p.o. for 11 weeks	No difference in urine tests	- eating less: ↑ - more energy: ↑ - drowsiness: ↓ - jitteriness: ↑ - ‘liking' (POMS): ↑ - blood pressure: ↑ - task performance: ↑ - craving: ↓↑	No
Levin et al. (194)	Titrated from 10 mg/day/p.o. to 40 mg/day p.o. to 80 mg/day p.o. in ADHD and opioid-dependent patients receiving methadone maintenance and 53% fulfilling cocaine dependence criteria	No difference in drug use	- compliance: ↓↑ - ADHD symptoms: ↓↑ - methadone maintenance: ↓↑ - fatigue: ↓↑ - sweating: ↓↑	No
Levin et al. (187)	Titrated from 10 mg/day/p.o. to 40 mg/day p.o. to 60 mg/day p.o. in cocaine-dependent ADHD patients for 14 weeks	Decreased probability of positive urine tests	- retention: ↓↑ - ADHD symptoms: ↓↑	Yes
Ling et al. (183)	Week 1: 18 mg/day p.o. Week 2: 36 mg/day p.o. Week 3-10: 54 mg/day p.o.	Fewer self-reported methamphetamine use days from baseline. No significant difference in number of positive urine drug screens at week 10, but less likely positive at week 14.	- cannabis positive drug screens: ↓ - craving: ↓ - retention: ↓↑ - adverse events: ↓↑ - treatment satisfaction: ↓↑	Yes
Miles et al. (184)	54 mg/day p.o. for 20 weeks	No different abstinence from methamphetamine rates compared with placebo	- retention: ↑ - craving: ↓↑ - severity of dependence: ↓↑	No
Minarik et al. (189)	Individual titration from 20 mg to 60 mg/day p.o. for 8 months	10 cases of abstinence out of 24 cases	- one case of alcohol poisoning - quality of life: ↑ - health conditions: ↑	Yes
Noroozi et al. (195)	60 mg/day p.o. for 12 weeks	No difference in negative urine tests	- craving: ↓↑ - withdrawal: ↓↑ - addiction severity: ↓↑ - depression: ↓↑ - high-risk behaviors: ↓↑	No
Rezaei et al. (185)	Week 1: 18 mg/day p.o.; Week 2: 36 mg/day p.o.; Week 3–10: 54 mg/day p.o.	Higher abstinence from methamphetamine rates	- craving: ↓ - depression score: ↓ - adverse events: ↓↑	Yes
Schubiner et al. (196)	Titrated from 30 mg/day p.o. to 60 mg/day p.o. to 90 mg/day p.o. for 12 weeks in cocaine users with ADHD	No difference in reported cocaine use or money spent on cocaine	- retention: ↓↑ - insomnia: ↑ - sadness: ↑ - single case of hypertension - single case of disorientation, insomnia, and anxiety - inattentive symptoms: ↓↑ - hyperactive symptoms: ↓ - craving: ↓↑	No
Tiihonen et al. (188)	Week 1: 18 mg/day p.o.; Week 2: 36 mg/day p.o.; Week 3–20: 54 mg/day p.o.	Fewer positive urine tests for amphetamine compared with placebo	- retention: ↓↑	Yes

↓, decrease; ↑, increase; ↓↑, mixed findings; p.o., per os; i.v., intravenous.

The neural mechanism from which these improvements may result remains unclear. Nonetheless, some data suggest that MPH reduces abnormally strong ventral to dorsal striatal functional connectivity in cocaine SUD patients, where addiction severity was associated with lower connectivity (197). These findings are in line with the prominent theory describing a ventral to dorsal striatal shift in the governance of behavior (33, 34). Another mechanism may be that MPH intervenes in assigning reward value to a drug experience. Evers et al. (198) observed that increased tonic dopamine activity by MPH reduced phasic ventral striatal response upon receiving reward. MPH may therefore suppress reward value and diminish reward-based learning in SUD patients, which is in line with an MPH-induced reduction in functional connectivity between the nucleus accumbens and ventral pallidum (199) as a neural substrate for drug liking (200). Improved behavioral control is suggested by MPH-induced altered functional connectivity between the nucleus accumbens medial PFC (199), an area associated with reflective cognition (201). In addition, MPH reversed an acute stress-induced reduction in brain activation associated with goal-directed behavior (202). Taken together, these data suggest MPH to increase behavioral control at times of choosing to perform a particular behavior associated with consequences including drug-related reward, an ability that may be key in maintaining abstinence.

### 2.5. Criticism

Despite the promising results, some criticism exists that needs to be considered. For example, cocaine users report increased drug wanting after MPH when prompted by relevant situations (203). MPH has also been shown to enhance the reinforcing effects of amphetamines in mice (204), and increase smoking behavior in neurotypical (205) and ADHD patients (206). Such effects would be counterproductive in a treatment setting. It should be noted that participants in both latter studies did not intend to quit smoking.

MPH-based treatment may also not be effective in some other addiction populations (186). For example, pathological gamblers have been shown to increase their motivation to gamble (207) and show increased DA release in dorsal striatal structures following amphetamine administration (208). The latter may contrast methamphetamine users in whom low levels of dorsal striatal DA release predict relapse (103) and in which MPH has shown to be effective the most. Another potential subgroup of SUD patients is formed by ADHD patients. There is a high comorbidity between ADHD and SUD (209, 210) for which various explanations exist. For example, individuals with undiagnosed ADHD may self-medicate with stimulants in an attempt to alleviate the symptoms (211). ADHD individuals also may be inherently vulnerable to SUD due to impulsive behavior and neurobiological characteristics (210). Subsequently, the proportion of ADHD patients in the SUD population is relatively large. The evidence for the efficacy of MPH to treat SUD in this particular population is limited, as many of these patients are already treated with MPH while SUD is still present [c.f. Wilens and Morrison (211)]. Crunelle et al. (89) detected that the limited success of MPH in cocaine-using ADHD patients compared with an ADHD-only group did not correlate with lower DAT occupancy. In addition, the effect of MPH is compromised in ADHD patients with comorbid cocaine dependence (196). On the other hand, MPH treatment of ADHD children has been shown to reduce the risk of substance (ab)use during adolescence (212). Therefore, individuals presenting with comorbidity at a later age may be predominantly more treatment resilient. It should be considered that the population with such comorbidity may require a different treatment approach.

Results of studies assessing the efficacy of MPH in the SUD population are not unequivocal (186), with some large, well-designed studies showing no difference between MPH treatment and placebo [e.g., Noroozi et al. (195)]. In addition, Dursteler et al. (182) report negative findings concerning the efficacy of MPH as a replacement medication in patients with cocaine use disorder specifically in five randomized controlled trials (187, 192–194, 196). However, these studies did not form a homogenous group. Differences between the studies exist as some studies included patients with ADHD and others did not. Other differences were the use of concomitant medication, duration of treatment, and dose of MPH. All the above criticisms could be considered a starting point for determining boundary conditions in which MPH may be effective; e.g., in which populations and with which doses may MPH be most effective?

Another potential limitation to the use of MPH as treatment is its abuse potential and associated health risk. Abuse potential may be suggested by observed behavioral cross-sensitization with amphetamine (213), increased drug-seeking behavior (204), and conditioned place preference (214) in rodents. The abuse potential may also be signaled by the abundant use among the student population (215), which may be associated with the risk of cardiac disease (216). Moreover, MPH has been ranked 12th on a list of substances causing physical harm (217). However, most studies in humans have not observed reinforcing effects of MPH using clinical oral doses [for an overview see Kapur (218)]. In addition, conditioned place preference for orally administered MPH was only observed for higher doses or when administered immediately before testing (214). The slow onset of effects of orally administered MPH [in contrast to intranasal administration (219)] and its slow clearance have also been associated with reduced abuse potential compared with that of other stimulants (220). In conclusion, misuse of MPH is observed and is associated with health risks. However, potential clinical use to treat SUD may be safe in clinical oral doses and when closely monitored to guard against abuse.

One of the pillars of the current argument is that hypofrontality is characteristic of impaired inhibitory control and is shared by ADHD and SUD. However, hypofrontality is not exclusive to ADHD and SUD but also occurs in schizophrenia (221), bipolar disorder (222), and major depressive disorder (223). Therefore, the fact that ADHD and SUD share this characteristic is insufficient to treat both with the same pharmacological agent, as this is clearly undesirable in, for example, schizophrenia. In addition, the functional interplay between frontal and striatal areas underlies working memory, attentional function, and task-switching performance (224) as well as response inhibition, which means that hypofrontality alone cannot be considered evidence of impaired inhibitory control specifically. Therefore, shared hypofrontality in isolation should not be considered conclusive evidence of similarities between the disorders in inhibitory control deficits. Instead, it should be considered in conjunction with the overlap in theoretical models, and behavioral and, most importantly, DA deficits.

## 3. Discussion

The primary objective of this paper was to explore shared behavioral and neurobiological atypicalities between ADHD and SUD to evaluate the potential usefulness of MPH treatment for SUD. Overlap between disorders can be considered based on their independent explanatory hypotheses stating impaired or biased cognitive control. Empirically, both show inhibitory behavior deficits as subjectively and objectively measured using the BIS-11, and SST, antisaccade, perseveration, conflicting motor response, and go/no-go paradigms. Functionally, they both show hyperactivation of the mesolimbic pathways, albeit for ADHD only in animal models. Atypical neurotransmission is shared by both disorders characterized by low tonic DA signaling and higher phasic response given appropriate stimuli. Finally, behavioral control networks, including frontal gyri, ACC, OFC, and DLPFC (53, 155), and frontal gray matter is prominently compromised in both disorders, which is in line with the behavioral deficits. These observations support the application of DA-based stimulants as a treatment for SUD, and current evidence identifies MPH as the main candidate among other stimulants.

Despite the shared characteristics, there are differences between ADHD and SUD patients. For example, a prominent ADHD symptom is inattentiveness, which is not commonly observed in SUD individuals (1). In addition, SUD individuals' impulsive behavior appears to be restricted to responding to drug-related cues, whereas ADHD patients behave disinhibited regardless of the context (60). For example, ADHD individuals show increased reward-circuit response during a monetary incentive task (112), while SUD patients often show a reduced response [e.g., Luijten et al. (225)]. ADHD patients commonly use compensatory strategies involving parietal attention areas to mask their cognitive deficits, while SUD patients do not show these additional activity patterns (53). Until now, only individuals with ADHD show unusual activity patterns in the DMN and the OFC is dysfunctional primarily in SUD patients (101, 151). Structural differences between these populations are also present with putamen having been shown to be enlarged (170) in SUD, but decreased in ADHD (28). Important for the present purpose of evaluating MPH suitability as a treatment for SUD, DA-related characteristics in ADHD appear to be mostly represented by high levels of DAT (85, 86), while results for SUD are inconsistent. As MPH blocks the DAT, similar characteristics may be desired. However, both conditions share low tonic DA and high phasic DA (79, 226) that may be reversed using MPH regardless of the DAT differences.

Future studies should be aimed at clarifying apparent discrepancies, behavioral relevance of neurobiological and neurofunctional atypicalities, and the effects of MPH on these measures of performance. For example, on a behavioral level studies may directly compare ADHD and SUD individuals on BIS-11 scores and SST performance. It may be established whether both populations differ to an equal extent from neurotypical, healthy controls. Performance on the SST may also be subject to boundary conditions. For example, it should be established whether SUD individuals only show higher SSRT when presented with addiction-relevant cues or whether they are impaired across a variety of task conditions. Such knowledge can aid in designing situation-specific treatments.

In addition to behavior, further direct comparisons between the populations should also be made concerning brain activation [e.g., Gerhardt et al. (78)]. The key questions that need to be answered are (1) whether ADHD and SUD individuals show similar altered responses of brain networks governing behavioral control, and if so, (2) are hypofunctional frontal brain areas equally relevant in both disorders for behavioral control specifically? (3) What is the contribution of other executive function deficits to the maintenance of the disorders? Multiple executive functions are impaired in both groups, e.g., reward processing, and further studies should establish similarities between the disorders and the effects of stimulant treatment of these functions (49). These functions can be assessed using various well-established performance tasks addressing different aspects of behavioral control like SST assessing motor control (42), gambling tasks assessing reward learning (227), and devaluation tasks assessing goal-directed behavior (228). Concerning the latter, and in parallel to the theory that addiction is characterized by a transition from goal-directed toward habitual behavior, a direct comparison would be very valuable to determine whether ADHD individuals show a similar shift in activation of brain areas governing goal-directed/habitual behavior as is often observed in SUD individuals [e.g., Sjoerds et al. (229)]. In addition, future studies may investigate whether factors that are known to elicit habitual behavior [e.g., stress, Schwabe and Wolf (230)] are equally effective in these populations in affecting brain activation and associated goal-directed behavior.

Further key questions concern explaining the inconsistent observations of DAT levels in SUD. For example, it may be argued that if DAT levels are a consequence of drug use, different drugs may affect DAT levels to a different extent. Drugs exerting their effects through strong activation of catecholaminergic neurotransmission (e.g., amphetamine, cocaine) may induce downregulation of DAT, while drugs like cannabis and heroin may do this to a lesser extent. Within that context, most evidence for the efficacy of MPH in SUD comes from studies in stimulant use disorder patients. It appears reasonable that SUD involving catecholaminergic drugs (e.g., cocaine, amphetamine) may benefit most from MPH treatment. It remains an empirical question whether all addictive disorders are equally suitable to be treated with MPH. It is assumed that all addictions share underlying neurobiological alterations, and based on that notion, it can be hypothesized that MPH is potentially effective in all forms of addiction. However, such extrapolation should be made carefully and awaits empirical confirmation, as most studies showing efficacy in SUD only concern stimulant use disorder patients.

If subpopulations within SUD can be identified, treatments may be tailored to these groups such that MPH treatment may be suitable for one but not the other group or that different dosages may be needed. As well as SUD subgroups, ADHD subgroups (inattentive, impulsive, combined) should be considered. It may be predicted that the impulsive type shares most behavioral and neurobiological characteristics compared with the inattentive type. However, the efficacy of MPH in potential subgroups remains an empirical question that future studies should answer.

Another characterization that may define a suitable sub-population of patients may be neuroimaging measures of the dopamine system. For example, cocaine-dependent patients that show high D2/3 receptor binding and dopamine release following MPH choose a monetary incentive over cocaine more often compared with patients showing less D2/3 binding and dopamine release (231). Such methods may even be hypothesized to assess sensitivity to treatment effects on an individual level. It has been argued that individual tonic dopamine levels are associated with the clinical effectiveness of treatments (21). Such variability in tonic dopamine levels can further be utilized to define individual treatment needs. For example, monetary rewards can be an effective reward for cocaine (231, 232) and smoking (233) abstinence. It is an outstanding hypothesis that the level of dopamine responding to MPH or D2/3 receptor binding can define the height of the monetary incentive, such that lower dopamine system level responding requires larger rewards. In conclusion, more detailed information concerning subgroups, individual differences, and other boundary conditions is needed to determine the suitability of MPH in treating SUD.

The current paper is limited in its scope in evaluating the potential use of MPH as treatment for SUD. Nonetheless, alternative approaches to treating SUD should be considered. Thus far, part of the rationale presented in the current paper to treat SUD patients with MPH is based on a current theoretical explanation of addiction (31, 32). Addiction-related cues elicit a DA response in the ventral striatum, which is associated with an intense craving (“wanting”) of the drug. MPH is theorized to be able to reduce the phasic DA response and therefore the craving. An alternative approach to the function of DA and the mesolimbic system is one in which the system provides a learning signal whenever a reward is larger than predicted [reward-prediction error; (234)]. This signal aims to strengthen the association between the stimulus, response, and outcome (235). In rodents (236) and humans (237, 238), high doses of nicotine have been observed to enhance this signal, which may consequently continue the learning of and strengthen the associations between smoking cigarettes and obtaining reward. An effective treatment may be the dampening of the DA signal whenever a cigarette is smoked to reduce the positive reward-prediction error. MPH has been shown to reduce ventral striatal activation in response to receiving a reward (198). In addition, psilocybin (a hallucinogenic substance found in “magic mushrooms”) may also reduce phasic DA neurotransmission in the mesolimbic pathway. Psilocybin is a 5-HT2A agonist, which predominantly is expressed in the mesolimbic pathways. There is consensus that 5-HT2A activation inhibits DA release (239). Therefore, it can be hypothesized that psilocybin administration leads to a reduced positive reward-prediction error and the association between substance taking and reward. Future, studies should test these hypotheses derived from this rationale.

Concerning alternative approaches, non-invasive brain stimulation or neurofeedback increases prefrontal activity, reduces impulsivity, and enhances cognitive functions (14, 88, 140) and may therefore be considered a potential treatment. In addition, for drug-dependent individuals it is important to train psychosocial skills alongside pharmacological treatment to ensure abstinence. Learning new coping skills, developing a new support system, and challenging expectations about drugs are important factors that enhance self-regulation abilities (156).

Taken together, this perspectives paper provides evidence toward a dimensional approach of psychopathology and serves as an illustration for the promise of developing transdiagnostic treatment programs. More specifically, it contributes to the development of novel pharmacological treatment approaches that may be based on treatments for disorders that are similar in underlying etiology (9). The transdiagnostic symptoms of disinhibition and impulsivity that are characteristic of both SUD and ADHD may have overlapping underlying etiology, namely, abnormal tonic/high phasic DA transmission that leads to a strong drive to perform a given action. This behavior may be associated with prefrontal hypoactivity and brain structural deficits. The DA transmission deficit can be treated with MPH, which has been proven successful in ADHD and may be suitable for use in SUD. The key question is whether the maladaptive behaviors in ADHD that can be treated with MPH are indeed resulting from the DA atypicalities that are shared by both conditions. Also, it is clear that ADHD and SUD are not the same, and it should be studied whether the neurobiological differences underlie other aspects of the respective phenotypes.

## Author contributions

PR and LF contributed to establish the first draft of the manuscript and contributed to developing the rationale. LF summarized the existing studies. PR wrote the first draft of the manuscript. NM, JR, and PS contributed to the manuscript in its current version. All authors contributed to the article and approved the submitted version.
